# TMPyP4, a Stabilizer of Nucleic Acid Secondary Structure, Is a Novel Acetylcholinesterase Inhibitor

**DOI:** 10.1371/journal.pone.0139167

**Published:** 2015-09-24

**Authors:** Nana Fujiwara, Michael Mazzola, Elizabeth Cai, Meng Wang, John W. Cave

**Affiliations:** 1 Burke Medical Research Institute, White Plains, New York, United States of America; 2 Weill Cornell Medical College, Brain and Mind Research Institute, New York, New York, United States of America; University of Colorado Denver, UNITED STATES

## Abstract

The porphyrin compound, TMPyP4 (5,10,15,20-Tetrakis-(N-methyl-4-pyridyl)porphine), is widely used as a photosensitizer and a modulator of nucleic acid secondary structure stability. Our group recently showed in cultured cells and forebrain slice cultures that this compound can also down regulate expression of *Tyrosine hydroxylase* (*Th*), which encodes the rate-limiting enzyme in catecholamine biosynthesis, by stabilizing DNA secondary structures in the *Th* proximal promoter. The current study sought to establish whether treatment with TMPyP4 could modify mouse *Th* expression levels *in vivo*. Intraperitoneal administration of low TMPyP4 doses (10mg/kg), similar to those used for photosensitization, did not significantly reduce *Th* transcript levels in several catecholaminergic regions. Administration of a high dose (40 mg/kg), similar to those used for tumor xenograph reduction, unexpectedly induced flaccid paralysis in an age and sex-dependent manner. *In vitro* analyses revealed that TMPyP4, but not putative metabolites, inhibited Acetylcholinesterase activity and pre-treatment of TMPyP4 with Hemeoxygenase-2 (HO-2) rescued Acetylcholinesterase function. Age-dependent differences in HO-2 expression levels may account for some of the variable *in vivo* effects of high TMPyP4 doses. Together, these studies indicate that only low doses of TMPyP4, such as those typically used for photosensitization, are well tolerated *in vivo*. Thus, despite its widespread use *in vitro*, TMPyP4 is not ideal for modifying neuronal gene expression *in vivo* by manipulating nucleic acid secondary structure stability, which highlights the need to identify more clinically suitable compounds that can modulate nucleic acid secondary structure and gene expression.

## Introduction

Nucleic acid secondary structures, such as G-quadruplexes and i-motifs, are widespread genomic regulatory elements capable of modulating both gene transcription and translation. *In silico* analyses suggest that transcription of approximately 40% of the human genome is regulated by G-quadruplex structures within the 1kb proximal promoter region [1]. This may be a significant underestimate, however, since about 375,000 potential G-quadruplex motifs are present in the entire genome [1, 2], and many of these motifs are in gene introns, exons and untranslated regions as well as gene proximal promoters. Thus, the roles of nucleic acid secondary structures as critical regulatory elements in gene expression are likely to be pervasive within both mammalian genomes and transcriptomes.

The biological significance of nucleic acid secondary structures has been extensively studied in cancer biology, where these structures are both integral to the maintenance of chromosomal telomeres and can form within oncogene proximal promoter regions to modulate transcription activity [3, 4]. Recent studies have shown that nucleic acid secondary structures also regulate neuronal gene expression. Our laboratory showed transcription of *Tyrosine hydroxylase* (*Th*), the rate-limiting enzyme for catecholamine neurotransmitter biosynthesis, is regulated by secondary structures in its proximal promoter region [5]. Others have shown that hexanucleotide expansions in a non-coding region of *C9orf72* enable the formation of G-quadruplexes in both the genomic DNA and transcript RNA that result in abortive transcription and the development of neurodegenerative disease pathologies.[6] Also, G-quadruplex formation in the untranslated region of *Post-synaptic density protein 95* (*PSD-95*), which is prone to trinucleotide-repeat expansion and pathogenic for Fragile X syndrome, regulates access of miR-125a and modulates translation of PSD-95 mRNA [7]. In addition to the nervous system, recent studies have also shown that transcription of genes essential for muscle, cranio-facial and cardiac development is regulated by secondary structures within the proximal promoter [8–10].

**Fig 1:**
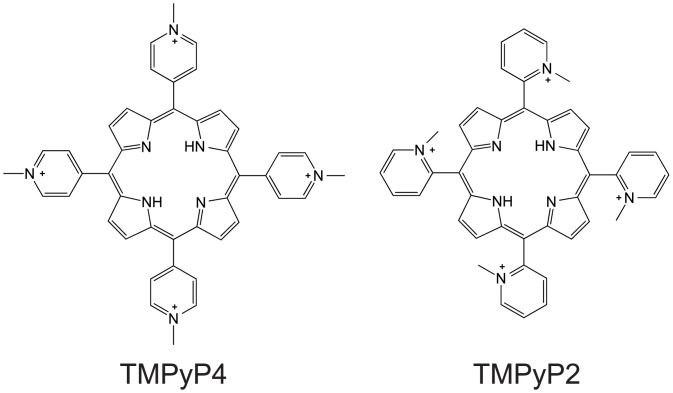
Molecular structures of TMPyP4 and TMPyP2.

Both the ability to modulate gene expression and the association with disease pathology, such as cancer and neurodegeneration, have generated interest in identifying pharmaceutical agents that modify nucleic acid secondary structural stability [11–14]. One of the most extensively used compounds to manipulate nucleic acid secondary structure stability is TMPyP4 (5,10,15,20-Tetrakis-(N-methyl-4-pyridyl)porphine; Fig 1). This porphyrin molecule stabilizes both G-quadruplexes and i-motifs, whereas its structural isomer, TMPyP2 (Fig 1), does not [15–17]. Many studies using cultured cells have demonstrated that TMPyP4 can reduce cell proliferation and stabilize nucleic acid secondary structure to modify gene transcription levels [18–24], but only a handful have studied this compound *in vivo* [23, 25–29]. These *in vivo* studies have primarily focused on the ability of TMPyP4 to reduce the size of tumors either by itself or in combination with photodynamic therapy rather than demonstrating that TMPyP4 can alter either gene transcription or translation levels *in vivo*.

In this report, we investigated whether *Th* expression levels *in vivo* could be modulated by intraperitoneal injection of TMPyP4 at doses comparable to those previously used for tumor reduction studies. These previous studies, in general, have tested either photoexcitation of TMPyP4 and the ability of TMPyP4 to remove tumor xenographs. The photoexcitation studies typically used lower dosages (approximately 10mg/kg), whereas xenograph studies often used higher doses (approximately 40mg/kg). The current studies also build on our previous studies showing that TMPyP4 reduced *Th* promoter activity in forebrain slice cultures [5], and reveal that low doses of TMPyP4 (10 mg/kg) did not significantly alter *Th* expression levels *in vivo*. Unexpectedly, administering a high dose (40 mg/kg) resulted in temporary flaccid paralysis that was age and sex-dependent. *In vitro* analyses revealed that TMPyP4, but not its putative metabolites, inhibited Acetylcholinesterase (AChE) activity and pre-treatment of TMPyP4 with hemeoxygenase-2 (HO-2) rescued AChE function. Age and sex-dependent differences in HO-2 expression levels may partly account for the variable *in vivo* effects of TMPyP4 observed. Together, these studies show that only low doses of TMPyP4, such as those typically used for photosensitization, are tolerated *in vivo*. Furthermore, despite its widespread use *in vitro*, TMPyP4 is not ideal for modifying neuronal gene expression *in vivo* by manipulating nucleic acid secondary structure stability, which highlights the need to identify more clinically suitable compounds that are.

## Materials and Methods

### Animals

All studies used adult transgenic mice expressing green fluorescent protein (GFP) under the control of the 9kb *Th* promoter.[30] Mice between 1.5 and 3 months of age were designated as young adults, whereas mice between 6 and 12 months of age were designated as old adults. All mice were housed in humidity-controlled cages at 22°C under a 12:12 h light/dark cycle and provided with food and water *ad libitum*. All animals were sacrificed by asphyxiation with carbon dioxide. All procedures were carried out under protocols approved by the Weill Cornell Medical College Institutional Animal Care and Use Committee and conformed to NIH guidelines.

For mice receiving low doses of TMPyP2 or TMPyP4, intraperitoneal injections of saline or 10mg/kg of either TMPyP2 (Frontier Scientific) or TMPyP4 (Sigma-Aldrich) were administered twice daily for 5 days. Eight adult animals (3–12 months of age) of each sex were used. Mice were euthanized 2 hours after the final injection and the adrenal glands, olfactory bulbs and midbrain were collected for analysis of *Th* transcript levels.

For mice receiving high doses of TMPyP4 or TMPyP2, single intraperitoneal injections of saline or 40mg/kg of either TMPyP2 or TMPyP4 were administered. The behavior of mice was monitored 15 minutes prior and every 15 minutes for 2 hours after injection. At each time point, mice were qualitatively evaluated for breathing rate, muscle tone, righting reflex and forepaw grasping. Three animals from each sex and age were used. Mice receiving single intraperitoneal injections of saline or 4-formyl-1-methylpyridinium benzenesulfonate hydrate (4F-MP; Alfa Aesar) or 4-carboxy-1-methylpyridinium (4C-MP; Sigma-Aldrich), were also monitored 15 minutes prior to injection and every 15 minutes for 2 hours after injection. At each time point, mice were qualitatively evaluated for breathing rate, muscle tone, righting reflex and forepaw grasping.

### Cell Culture

Human SH-SY5Y neuroblastoma cells were maintained at 37°C with 95% air and 5% CO_2_ in DMEM/F12 media supplemented with plus GlutaMax (Life Technologies). For transcription assays, cells were plated on Primaria 6-well plates, and Lipofectamine LTX transfection reagent (Life Technologies) was used to transfect 1.5 μg of a pGL4.20 luciferase reporter plasmid containing the 4.5kb upstream region of the rat *Th* promoter. At the time of transfection, cells were also treated with either saline or 100 μM of either TMPyP2 or TMPyP4. After 24 hours, cells were harvested and luciferase activity levels were measured using the firefly luciferase assay kit (Promega). Luminescence was measured with LMaxII luminometer (Molecular Devices). Luciferase activities are reported as the mean of at least three independent measurements with error bars representing the standard deviation.

### Quantitative RT-PCR

Total RNA was isolated from 30mg of both liver and rear leg skeletal muscle of mice using the RNeasy Mini Kit (Qiagen) and following the manufacturer’s instructions. First-strand cDNA syntheses were generated with SuperScript™ II Reverse Transcriptase (Invitrogen). Quantitative PCR reactions were performed with TaqMan assays (Applied Biosystems) for *Th* (Mm00447557_m1), *beta-Actin* (4352341E), beta-tubulin III (Mm00727586_s1) and GAPDH (Mm99999915_g1) with TaqMan Universal PCR Master Mix (Applied Biosystems). All reactions were carried out on a 7500 fast real-time PCR System (Applied Biosystems). Expression levels for *Th* were normalized to those of *beta-Actin*, beta-tubulin III and GAPDH. All samples were run in triplicate. The mean relative expression levels are reported with error bars representing the standard error of the mean. Statistical significance was analyzed by ANOVA with Tukey’s post-hoc test using Prism software (GraphPad).

### AChE Activity Assays

AChE activity was measured a fluorometric assay kit (Abcam) with a Gemini EM Fluorescence microplate reader (Molecular Devices) and SoftMax Pro software (Molecular Devices). Assays to test for AChE inhibition used 10nM, 100nM, 1μM, 10μM, and 100μM of the following inhibitors: TMPyP2 (Frontier Scientific), TMPyP4 (Sigma-Aldrich), Donepezil hydrochloride monohydrate (Sigma-Aldrich), 4-formyl-1-methylpyridinium benzenesulfonate hydrate (4F-MP; Alfa Aesar) and 4-carboxy-1-methylpyridinium (4C-MP; Sigma-Aldrich). AChE activities are reported as a fraction of the activity observed with 50μU of an AChE standard supplied with the fluorometric assay kit. For each concentration, AChE activities are reported as the mean of 3 independent trials with error bars representing the standard error of the mean.

For experiments to test whether pre-treatment with HO-2 could block TMPyP4-mediated inhibition of AChE activity, stock solutions of 0.5mg/ml of TMPyP4 were incubated with 0.05, 0.1, 0.5, or 1.0 μg of recombinant rat HO-2 (Enzo Life Sciences) at room temperature for 15 minutes. Following this incubation, the solution was heat-inactivated for 5 minutes at 65°C, and then cooled to 25°C before being added to the AChE assay so that the final concentration was 25μM TMPyP4. AChE activities are reported as the mean activity from 3 independent trials with error bars representing the standard error of the mean.

For the determination of AChE activity in liver and skeletal muscle of young adult male and female mice, 20–40mg of tissue samples were flash-frozen in liquid nitrogen, crushed with a pestle and then resuspended in 0.1M PBS with protease inhibitor cocktail (Thermo Scientific) before being sonicated. Tissue samples were collected from 2 young adults of each sex. Total protein concentration of tissue lysates was determined by a Bradford assay kit (Bio-Rad), which was used to normalize all AChE activity from the respective tissue. The normalized AChE activities are reported as a fraction of the activity observed with 50μU of an AChE standard supplied with the fluorometric assay kit. Linear regression of the relative AChE activity as a function of tissue lysate concentration was performed with Prism software (GraphPad). The slopes of linear regressions were analyzed for significant differences by an ANCOVA-like algorithm in the Prism software package.

### Western blot analysis

Liver and rear leg skeletal muscle samples were obtained from 3 animals of each sex and age. Samples were frozen in liquid nitrogen and homogenized in PBS and Laemmli buffer at 1:1 dilution. 160μg of homogenized lysate was added to 2x Laemmli buffer at a 1:2 dilution, then loaded and resolved on a NuPAGE® Novex® 4–12% Bis-Tris Gel (Life Technologies) before being transferred to membrane. Proteins were visualized with a rabbit anti-GAPDH antibody (Sigma-Aldrich) at 1:5000, rabbit anti-HO-2 antibody (Enzo Life Sciences) at 1:500, and rabbit anti-HO-1 antibody (Enzo Life Sciences) at 1:500. Goat anti-rabbit IR680 or IR800 fluorescent secondary antibody (Li-Cor Biosciences) was used at 1:5000. Membranes were visualized using an Odyssey imaging system (Li-Cor Biosciences) and band intensities were quantified using ImageStudio Lite software (Li-Cor Biosciences). All HO-1 and HO-2 protein levels were normalized to corresponding GAPDH levels. Graphs report the mean normalized protein levels with error bars representing the standard error of the mean. Statistical significance was analyzed by ANOVA with Tukey’s post-hoc test using Prism software (GraphPad).

## Results

### In vivo Th expression levels in mice administered low doses of TMPyP4

**Fig 2:**
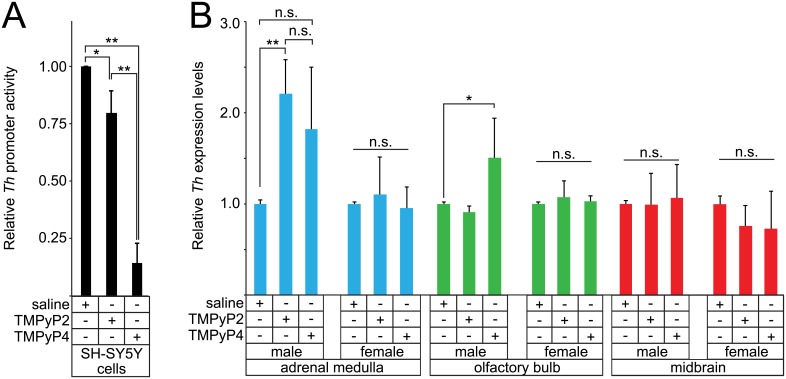
*In vitro vs*. *in vivo* modulation of *Th* expression by TMPyP4. **A**, transcription assays with SH-SY5Y cells transfected with a reporter under the control of the rat 4.5kb *Th* promoter that are also treated with either saline, TMPyP2 or TMPyP4. Mean relative promoter activities are shown with error bars representing the standard deviation. ** indicates p<0.001, * indicates p = 0.02 and n.s. indicates not significant. **B**, *Th* transcript levels, as measured by quantitative RT-PCR, in the adrenal glands, olfactory bulb and midbrain of adult mice administered either saline, TMPyP2 or TMPyP4 (n = 8 for both male and female). Mean relative transcript levels are shown with error bars representing the standard error of the mean. ** indicates p<0.01, * indicates p = 0.03 and n.s. indicates not significant.

We previously showed that the *Th* promoter can form G-quadruplex and i-motif secondary structures and assays with forebrain organotypic slice cultures indicated that stabilization of these secondary structures with TMPyP4 reduces *Th* promoter activity [5]. Transcription assays with SH-SY5Y cells transfected with a reporter containing the 4.5kb rat *Th* promoter and treated with either TMPyP2 or TMPyP4 confirmed that TMPyP4 can selectively repress *Th* promoter activity (Fig 2A).

To establish whether TMPyP4 can alter *Th* transcription levels *in vivo*, adult male and female mice (3–12 months of age) were administered saline or 10mg/kg of either TMPyP2 or TMPyP4 by intraperitoneal injection twice daily for 5 days. This low dosage was similar to those used in photosensitization studies [28, 29]. Two hours after the final injection, total RNA was prepared from the olfactory bulbs, midbrain and adrenal glands, all of which contain catecholaminergic neurons that express *Th*. Using *β-Actin* for reference, quantitative RT-PCR analysis of *Th* transcript levels revealed no significant changes in midbrain *Th* expression levels after exposure to TMPyP4 (Fig 2B). By contrast, the male olfactory bulbs showed a modest increase in *Th* expression levels (p = 0.03). The female olfactory bulbs, however, did not show any significant change. *Th* levels in the male adrenal glands also showed an increase (p = 0.05) in males treated with TMPyP4. This effect was not specific to TMPyP4 exposure, however, since TMPyP2 administration produced a similar increase. Using either β-Tubulin III or GAPDH as alternative reference genes did not alter the relative expression levels of *Th* in any of the tissue studied (data not shown). Together, these findings showed that low doses of TMPyP4 (≤10mg/kg) are, in general, not effective in modulating *Th* transcription levels *in vivo*.

### High doses of TMPyP4 induce temporary flaccid paralysis

To test whether higher doses of TMPyP4 were able to more robustly and significantly alter *Th* transcription levels *in vivo*, adult mice were administered either saline or 40mg/kg of either TMPyP4 or TMPyP2 by intraperitoneal injection. This higher dosage was similar to those previously used for reducing xenograph tumors [23]. Administration of the higher doses was planned to be the same as the lower doses (i.e., twice daily for 5 days). After the second injection, however, substantial mortality was observed in the group treated with TMPyP4. Close observation of mice receiving a single higher dose of TMPyP4 revealed that approximately 10 minutes after injection mice showed a reduced breathing rate and loss of muscle tone as well as grasping and righting reflexes. This flaccid paralytic state persisted for approximately 1–1.5 hours before the mice regained their normal breathing rates, muscle tone and reflexes.

Surprisingly, some animals were resistant to the effects of TMPyP4. Further analysis revealed that young adult male mice (aged 1.5–3 months) showed only a slight impairment of their righting reflex for approximately an hour, but otherwise displayed no other adverse symptoms. By contrast, older males (aged 6–12 months) as well as both young and old female mice all showed signs of flaccid paralysis following exposure to the higher dose of TMPyP4.

In contrast to TMPyP4, all mice treated with TMPyP2 had only a slight impairment in their breathing rate that persisted for about half an hour, but otherwise they did not show any signs of reduced muscle tone or grasping and righting reflexes. Table 1 summarizes the age- and gender-dependent physiological effects observed with a single higher dose of either TMPyP2 or TMPyP4.

**Table 1:** Physiological symptoms observed following a single injection with a high dose (40mg/kg) of either TMPyP2 or TMPyP4.

Symptom	Treatment	Young Males	Young Females	Old Males	Old Females
Breathing Rate	TMPyP4	++	+/-	+/-	+/-
	TMPyP2	++	+	+	+
Muscle Tone	TMPyP4	++	+/-	+/-	+/-
	TMPyP2	++	++	++	++
Grasping Reflex	TMPyP4	++	+/-	+/-	+/-
	TMPyP2	++	++	++	++
Righting Reflex	TMPyP4	+	-	-	-
	TMPyP2	++	++	++	++

Young adult mice were aged between 1.5–3 months, whereas old adults were aged between 6–12 months. Breathing rate, muscle tone, grasping and righting reflexes were qualitatively assessed: ++, normal/unaffected; +, moderately impaired; +/-, severely impaired;-, completely impaired. For each age and gender, n = 5 mice.

### TMPyP4 is an AChE inhibitor that is blocked by HO

**Fig 3:**
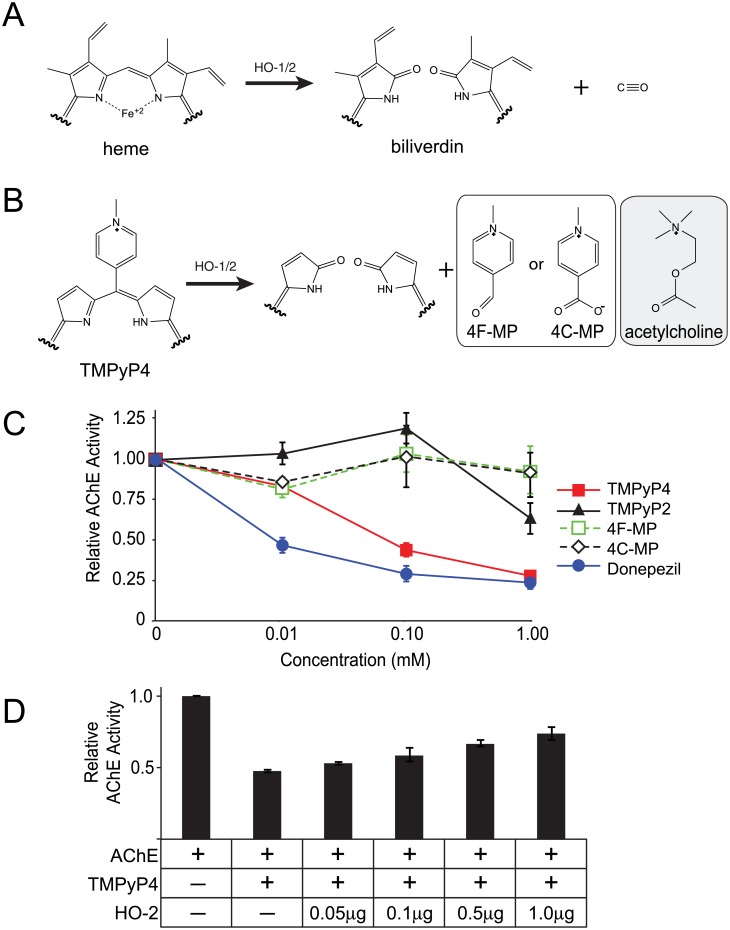
TMPyP4 inhibits AChE activity. **A**, oxidation of heme by Hemeoxygenase (HO) enzymes produces biliverdin and carbon monoxide. **B**, oxidation of TMPyP4 by HO enzymes is expected to generate either 4-formyl-1-methylpyridinium (4F-MP) or 4-carboxy-1-methylpyridinium (4C-MP). Both 4F-MP and 4C-MP have a structural resemblance to acetylcholine. **C**, fluorometric assay of AChE activity in presence of TMPyP2, TMPyP4, 4F-MP, 4C-MP and an established AChE inhibitor (Donepezil). **D**, pre-incubation of TMPyP4 with recombinant HO-2 prior rescued AChE activity in a concentration-dependent manner.

Given the symptoms observed following exposure to the higher dose of TMPyP4, we addressed whether TMPyP4 or one of its putative metabolic products impaired AChE, which degrades the neurotransmitter acetylcholine within neuromuscular junctions and is essential for control of muscular function. Porphyrin compounds are typically oxidized by either of two HO isoforms. HO-1 is an inducible isoform and HO-2 is a constitutively and widely expressed enzyme. Metabolism of heme by HO-1/2 generates carbon monoxide and biliverdin (Fig 3A). Based on this mechanism, oxidation of TMPyP4 by HO enzymes would be expected to generate either 4F-MP or 4C-MP (Fig 3B). Both of these putative metabolites resemble acetylcholine and may bind the AChE binding site.

Using a fluorometric AChE activity assay, we tested whether TMPyP4, 4F-MP or 4C-MP impaired AChE activity. As shown in Fig 3C, neither 4F-MP nor 4C-MP inhibited AChE activity. By contrast TMPyP4 was an effective AChE inhibitor, although it was not as potent as the established inhibitor Donepezil. Since TMPyP4 could inhibit AChE, but 4F-MP and 4C-MP could not, this suggested that HO-mediated breakdown of TMPyP4 could rescue AChE from TMPyP4-mediated inhibition. To test this possibility, TMPyP4 was incubated with HO-2 for 15 minutes at room temperature prior to being used in the fluorometric AChE reaction assay. Pre-incubation with HO-2, rather than direct addition of HO-2 with TMPyP4 to the assay reaction with AChE, was required because HO-2 degraded AChE under the assay conditions (data not shown). These studies showed that HO-2 rescued AChE activity from TMPyP4-mediated inhibition in a concentration-dependent manner (Fig 3D). Together, these findings show that TMPyP4, but not its putative metabolites, inhibits AChE. Consistent with this finding, single intraperitoneal injections with either 4F-MP or 4C-MP (6.4 and 10.2 mg/kg, respectively, which provided the molar equivalent 40 mg/kg TMPyP4) did not induce any of the adverse physiological effects observed with the high dose of TMPyP4 (data not shown).

### Age and gender-dependent distribution of HO-2

**Fig 4:**
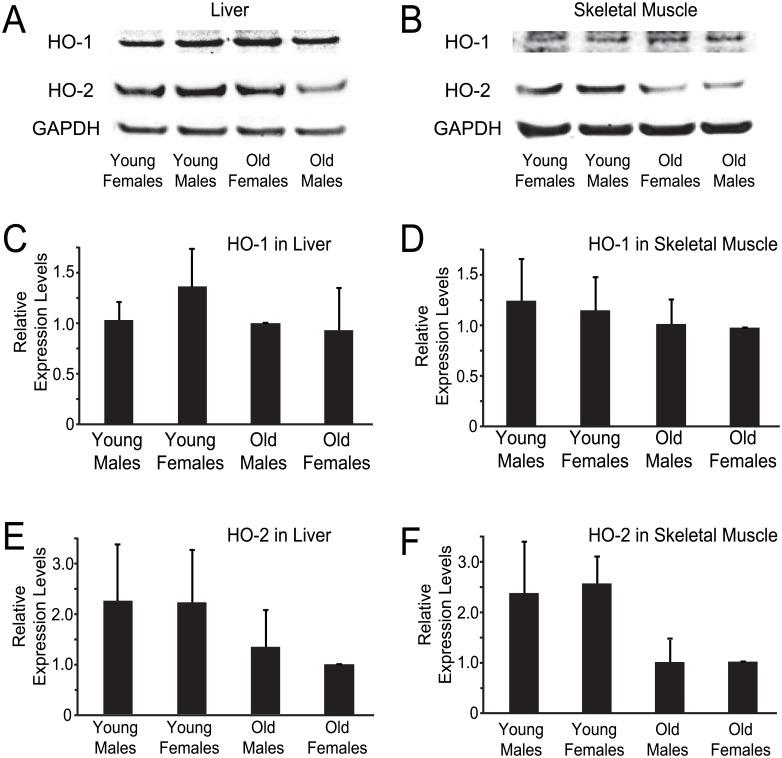
Age and gender analysis of HO-1/2 expression levels in liver and skeletal muscle. **A**-**B**, western blots for HO-1/2 and GAPDH in the liver and skeletal muscle, respectively, of young and old adult mice for both sexes. **C** and **E**, **D** and **F**, average relative western blot band intensities for HO-1/2 in liver and skeletal muscle tissue, respectively. Averages are from 3 independent trials for each age and gender in both liver and muscle tissue.

To determine whether age- or gender-based differences in the expression levels of either HO-1 or HO-2 could contribute the differential responses to TMPyP4, we quantified HO-1 and HO-2 protein levels from Western blots of liver and skeletal muscle lysates from both young and old adult mice. These studies found no differences in HO-1 protein levels in either liver or skeletal muscle tissue (Fig 4A–4D). By contrast, HO-2 protein levels in both the liver and skeletal muscle showed a trend as being greater in the young adult mice as compared to the older mice (Fig 4E–4H). This trend did not reach a level of significance (p>0.05), but the results suggest that there may be subtle differences in HO-2 expression levels between older and younger adults that may, in part, underlie the differential response to TMPyP4.

**Fig 5:**
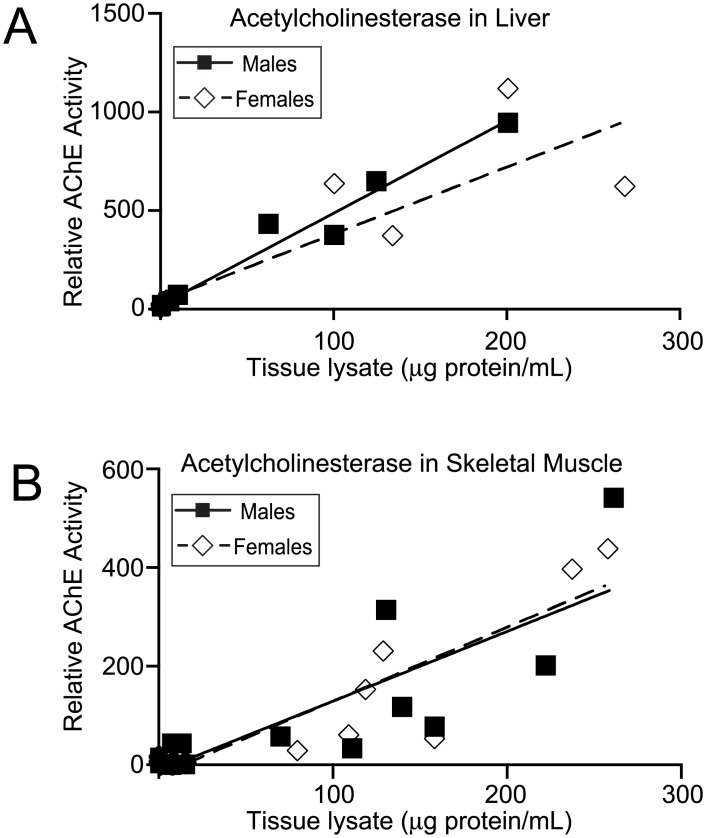
Gender-based analysis of AChE activity. **A** and **B**, fluorometric AChE activity assays with liver and skeletal muscle tissue lysates, respectively, from young adult mice (aged 1.5–3 months). For both males and females, data points are the combination of four individuals. AChE activities of tissue lysates are reported relative to the activity of an AChE standard supplied with the assay kit.

To establish whether inherent differences in AChE activity could underlie the gender-dependent sensitivities to TMPyP4, we measured AChE activity in liver and skeletal muscle lysates from young adult mice (Fig 5). These studies, however, showed no significant gender-based difference in AChE activity in either tissue.

## Discussion

The current study showed that TMPyP4 was ineffective as an *in vivo* modulator of *Th* expression levels. Low levels of TMPyP4 (10mg/kg), similar to those used for photosensitization studies, did not produce any adverse side effects, but they also did not significantly alter *Th* levels in most catecholaminergic neuronal regions examined. The male olfactory bulb, which has a dopaminergic interneuron subpopulation, did show a modest increase in *Th* transcript levels. This increase in *Th* levels was unexpected since stabilization of DNA secondary structure by TMPyP4 repressed *Th* promoter activity in cell and slice culture model systems [5]. The significance of the sensitivity of the male olfactory bulb dopaminergic neurons to either TMPyP4 or its metabolites is not clear since there are no known sex-dependent differences in either the function or development for these neurons. Attempts to use a higher dosage (40mg/kg) on a similar administration schedule in order to more robustly alter *Th* levels were thwarted by unanticipated toxic side effects (discussed further, below). Application of a single high dose, however, also did not alter *Th* expression levels (data not shown).

Previous studies showed that TMPyP4 can successfully reduce tumor xenograph size *in vivo* [23]. The mechanism of action for this tumor reduction is thought to be mediated, in part, by down-regulating the expression of some oncogenes through the stabilization of secondary structures in the gene promoter regions. TMPyP4 may be effective in stabilizing nucleic acid secondary structure and modifying oncogene expression in tumors because previous pharmacokinetic studies have shown that it preferentially accumulates in tumors as compared to most normal tissues [31]. These previous pharmacokinetic studies also found that TMPyP4 does not accumulate in the brain, suggesting that TMPyP4 does not cross the blood-brain barrier. An inability to penetrate this barrier could explain the inability of TMPyP4 to repress *Th* levels in the midbrain or olfactory bulbs. The *Th*-expressing cells in the adrenal gland are not protected by the blood-brain barrier, however, and changes in *Th* levels specifically attributable to TMPyP4 exposure were not seen in this current study. Thus, low doses of TMPyP4 are not effective for modulating *Th in vivo*.

A second unexpected finding in this study was the temporary state of impaired breathing and flaccid paralysis induced by a single high dose (40mg/kg) injection of TMPyP4. Our studies indicate that this paralysis was mediated, at least in part, by inhibition of AChE by TMPyP4. Since AChE is essential for degrading acetylcholine in the neuromuscular junction, AChE inhibition leads to an excess of acetylcholine and an over-stimulation of the post-synaptic acetylcholine receptors. This situation leads to cholinergic crisis, which is characterized by labored breathing and flaccid paralysis. Other studies have administered similar doses of TMPyP4, but have not reported these adverse effects [23, 26]. One of these studies did not use the mice until 7 hours after injection, which is long after the temporary effects of impaired breathing and flaccid paralysis would have resolved [26]. The other study administered 40mg/kg injections of TMPyP4 mice on a once-a-day schedule [23]. Our initial studies found that a second injection of 40mg/kg TMPyP4 within 12 hours was typically fatal, but waiting at least 24 hours between injections may have provided enough time for mice to more fully recover and avoid fatality from subsequent injections.

The temporary state of impaired breathing and flaccid paralysis induced by TMPyP4 was also age- and sex-dependent. Our studies show that HO-2 can block TMPyP4-mediated inhibition of AChE, and that there may be age-dependent differences in HO-2 expression levels that can partially explain these variable responses to TMPyP4. The mechanisms underlying the gender dependence, however, remain to be established.

To our knowledge, this is the first report of TMPyP4 as an AChE inhibitor, which is surprising since TMPyP4 is structurally distinct from other known AChE inhibitors [32]. The molecular mechanism of this inhibition is unknown, but the positively charged nitrogen of the methyl pyridinium group is reminiscent of the charged quaternary amine of acetylcholine and suggests that TMPyP4 interacts with the AChE active site. Based on a structural resemblance to acetylcholine, the putative oxidative degradation products 4F-MP and 4C-MP were expected to be active inhibitory compounds. Our studies, however, clearly showed that neither of these compounds disrupted AChE activity and that the intact TMPyP4 molecule was a far more potent inhibitor. Given that the AChE active site is buried at the bottom of a gorge lined with several hydrophobic side chains [33], it may be that part of the TMPyP4 porphyrin ring favorably interacts with these hydrophobic side-chains to stabilize the interaction. Additional structural studies are required to address the molecular interaction with TMPyP4 and AChE in order to elucidate the mechanism of inhibition.

Thus, despite the widespread use *in vitro*, these studies show that TMPyP4 is not ideal for modifying neuronal gene expression *in vivo* by manipulating nucleic acid secondary structure stability. By contrast, low doses of TMPyP4 may be suitable for anti-cancer strategies that either use photosensitization or target nucleic acid secondary structure stability. Given the emerging understanding of how nucleic acid secondary structure regulates gene expression in the nervous system, as well as the potential implications of using small molecules to regulate the expression genes through these structures, these findings highlight the need to identify clinically safe and effective compounds that can modulate gene expression through nucleic acid secondary structure.
